# Driving Under the Influence: An Unusual Case of Basal Ganglia Stroke Misdiagnosed as Intoxication

**DOI:** 10.7759/cureus.48097

**Published:** 2023-11-01

**Authors:** Faiza Butt, Kulsoom Durrani, Muhammad N Khan, Abdul Waheed

**Affiliations:** 1 Family Medicine, WellSpan Good Samaritan Hospital, Lebanon, USA; 2 College of Medicine, Penn State University, Hershey, USA; 3 Family Medicine, Wellspan Good Samaritan Hospital, Lebanon, USA; 4 Family and Community Medicine, Penn State University College of Medicine, Hershey, USA

**Keywords:** hemiballismus, positive symptoms, negative symptoms, residual hemiparesis, stroke, ballismus

## Abstract

Traditionally, strokes are characterized by negative symptoms, including contralateral hemiparesis, facial paralysis, and sensory loss in the upper face and upper extremities. Strokes rarely cause movement disorders such as ballismus, a severe chorea characterized by brief, sudden dance-like movements. Early identification of non-traditional stroke symptoms and risk factors for cerebrovascular disease is vital in providing timely treatment and improving patient outcomes. Our case highlights an uncommon complication of stroke and the need to use advanced imaging modalities, including MRI, to identify brain lesions when other testing is negative. This report adds to the body of literature highlighting ballismus, a rare presentation of stroke, and the lessons learned from managing this case.

## Introduction

Ischemic strokes, a complication of cerebrovascular disease, occur when arterial supply to the brain is acutely disrupted because of thromboembolism. This results in various symptoms depending on which area of the brain or brainstem is affected [1]. The middle cerebral artery (MCA) is the most common vessel in stroke pathology. MCA strokes are traditionally characterized by ‘negative’ symptoms, including contralateral hemiparesis, facial paralysis, and sensory loss in the upper face and upper extremities [1]. Presentation of cerebrovascular disease with ‘positive’ symptoms is rare but can be a potential complication of ischemic strokes affecting the basal ganglia structures involved in the inhibitory pathways [2]. This may present as ballismus, a rare hyperkinetic movement disorder characterized by involuntary, intermittent ballistic, wide amplitude movements involving the extremities. Ballismus is considered a severe form of chorea, characterized by brief, sudden, spontaneous, and dance-like movements on one side of the body [2]. Basal ganglia strokes are rare, with an incidence of about 1%. Development of hemiballismus post-stroke occurs in about 0.4 to 0.54% of acute stroke cases [3]. Thus, ballismus or hemiballismus (ballismus that only affects the ipsilateral extremities) due to stroke is an obscure presentation of ischemic strokes. This report presents a case of ischemic stroke causing hemiballismus, which was confused as intoxication.

## Case presentation

A 59-year-old female was brought to the WellSpan Good Samaritan Emergency Department by law enforcement officials after a traffic stop due to suspicion of intoxication. During the incident, the patient was given a breathalyzer test that resulted in 0.0% blood-alcohol content, and she was subsequently brought to the ED for her symptoms. On presentation to the ED, the patient was hypertensive with a blood pressure of 158/82 mmHg and was hemodynamically stable. She did appear to be in significant emotional distress and had constant, uncontrollable jerking movements of her left arm and leg which started 12-14 hours before presentation.

Further investigation revealed that the patient had a history of hypertension, hyperlipidemia, major depressive disorder, generalized anxiety disorder, and a recent traumatic divorce. Her medication list consisted of diltiazem, losartan, paroxetine, bupropion and trazodone. Additionally, she said she had been taking paroxetine for several years, and there was a recent increase in her bupropion and trazodone dosages due to the acute stress caused by her divorce. She denied any tobacco or alcohol use and disclosed occasional marijuana use. Family history was relevant for a deceased father with type 2 diabetes mellitus, a living mother with major depressive disorder, and a healthy adopted daughter. On review of systems, she was negative for all findings except abnormal movements of the left arm and left leg, agitation, decreased concentration, dysphoric mood, anxiety, and nervousness.

On exam, the patient was in acute distress, which resulted in her attempting to remove her IV. She was also tearful, had tangential thoughts, decreased concentration, and left-sided hemiballismus. Her National Institutes of Health Stroke Scale (NIHSS) was a 2 related to limb ataxia, indicating that the patient may have suffered a minor stroke. Laboratory investigation revealed unremarkable complete blood count (CBC), comprehensive metabolic panel (CMP), troponin, creatine kinase (CK), erythrocyte sedimentation rate (ESR), antinuclear antibody (ANA), acetaminophen, salicylate, urinalysis, and thyroid-stimulating hormone (TSH) tests. The lipid panel revealed 71 mg/dL triglycerides, total cholesterol of 244 mg/dL, high-density lipoprotein (HDL) of 66 mg/dL, and low-density lipoprotein (LDL) of 164 mg/dL. Urine toxicology was positive for cannabinoids, consistent with the information the patient disclosed.

Due to the patient’s history and her presentation, including laboratory and imaging findings, the initial differential included conversion disorder, psychosis, extrapyramidal symptoms from psychiatric medications, and cerebrovascular accident (CVA, stroke) involving the basal ganglia.

Because of the concern for stroke, computed tomography (CT) and computed tomography angiography (CTA) imaging of the head and neck were ordered and revealed no acute intracranial hemorrhage or mass effect. However, there was some crowding of the cerebellar tonsils at the craniovertebral junction and foramen magnum.

**Figure 1:**
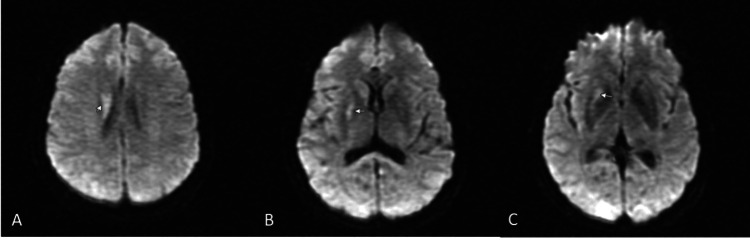
Brain MRI images with findings marked with arrows (A) A focus of increased signal intensity was noted in the body of the caudate nucleus adjacent to the body of the right lateral ventricle. This is increased in signal intensity on the T1-weighted image and low in signal intensity on the apparent diffusion coefficient (ADC) map, suggesting acute to subacute infarct. (B, C) A similar finding is visualized in the right lentiform nucleus with a mildly increased signal on the diffusion-weighted images, which is hypo-intense on the ADC map and consistent with an acute to subacute infarct.

Psychiatry was consulted and recommended that medication side effects and conversion disorder were unlikely in this case. The patient was started on the stroke optimization protocol which included starting aspirin, statin for goal LDL <70, permissive hypertension goal 140-180 systolic, O2 sat >94%, transthoracic echo with bubble study which was normal, and MRI brain without contrast. The MRI performed 24 hours after arrival to ED revealed the presence of an acute to subacute infarct in the body of the right caudate nucleus and the right lentiform nucleus (Figure 1).

During her hospital course, the patient was given paroxetine for her anxiety and depression while the bupropion and trazodone were held. Additionally, the patient was initially treated with haloperidol (Haldol®) 1-2mg every four hours as needed and was started on olanzapine (Zyprexa®) 2.5mg nightly dose for her acute agitation.

After stabilization, occupational and physical therapy were consulted and recommended discharge to home with home occupational therapy services. After a discussion of the prognosis of her condition, the patient was discharged on day 5 of hospitalization. No follow-up MRI was done on discharge. During her course, she showed significant improvement in her symptoms. Paroxetine was continued on discharge whereas trazodone and bupropion were discontinued.

## Discussion

Chorea is an involuntary movement disorder characterized by brief, sudden, spontaneous, and dance-like movements on one side of the body. Hemiballismus is considered a severe form of chorea [2]. Underlying causes of hemiballismus are varied and can include neoplasms, stroke, head trauma, metabolic causes, neuro-infections, and toxic exposures [2]. Due to the variety of underlying causes of hemiballismus, in the acute setting, it can be challenging to rapidly distinguish between the possibilities and evaluate for rare but time-dependent diagnoses such as stroke. In this case, the patient presented with a complicated social and psychiatric history and acute emotional distress that raised suspicion for psychiatric or medication-related pathologies in the initial differential diagnosis. However, stroke could not be ruled out.

Additionally, this patient was 59 at presentation. While the risk of stroke increases with age and over 70% of all strokes occur in patients older than 65, younger patients, especially those with significant risk factors such as coronary artery disease, hypertension, or diabetes mellitus, are not excluded from developing or suffering from complications of cerebrovascular disease [4]. Chung et al. reported on 27 patients who developed hemichorea after stroke and found that the average age of their cohort was 63 +/- 10 years of age [5], which is consistent with our case report. In contrast, Alarcón et al. and Pareés et al. reported older average ages for their populations at 74.5 and 73 years, respectively [6,7].

In our case, the patient also presented with classic vascular risk factors, including hypertension and hyperlipidemia, consistent with risk factors for cerebrovascular disease identified by Alarcón et al. We identified these vascular risk factors and this patient's potential cardiovascular accident. Consequently, we continued to include cardiovascular accidents as part of the differential diagnosis for this patient throughout the initial workup.

Initial evaluation with CT and CTA helped rule out hemorrhage; however, they could not identify the ischemic infarcts in the basal ganglia. These findings are consistent with the current understanding of both imaging modalities, as MRI is generally more sensitive than CT in detecting ischemia [8]. Two other studies that explored post-stroke hemiballismus cases similarly utilized MRI to identify the lesion, further emphasizing the difficulties of using CT to identify this type of lesion [9,10]. While the classification of the exact location of the stroke helps understand prognosis and symptoms, it should not delay treatment for prevention of further complications and treatment of the acute event.

Lenticular lesions and ischemic strokes most commonly result in hemiballismus development post-stroke, consistent with our findings [5-7]. Additionally, Chung et al. also found that hemiballismus usually develops on the day of stroke onset or within a few days and typically affects the contralateral leg and arm [5]. These findings were consistent with our case, where the patient presented with symptoms soon after the stroke based on imaging findings in the workup.

Patients with hemiballismus require treatment for the underlying cause of the movement and the movements themselves. Pharmacological treatment of the movement usually involves anti-dopaminergic drugs like haloperidol if symptoms are severe [5,7,10,11]. Most cases of hemiballismus spontaneously resolve, and the prognosis is good over the long term. In Chung et al. and Pareés et al., about 56% and 73% of patients with hemi-chorea, respectively, had resolution of their symptoms. For patients where it persisted, there was a decrease in the severity of symptoms over time in both studies [5,7]. Our findings are consistent with these results, as the patient was treated with haloperidol and olanzapine for her hospital stay. The haloperidol was discontinued on hospital discharge, and the olanzapine was continued daily. Subsequent follow-ups with the patient’s neurologist and family medicine provider demonstrated significant improvement in her symptoms.

Our case adds to the limited evidence that helps define uncommon stroke presentations. Further investigation is needed to continue to define these presentations, as prognosis largely depends on the timeliness of treatment and prevention of future events.

## Conclusions

Cerebrovascular accidents affecting the basal ganglia must be included in the differential diagnosis in patients presenting with unilateral, uncontrollable movement of the extremities. While cerebrovascular accidents are traditionally taught to present negative symptoms, presentation with positive symptoms, such as hemiballismus, is possible. These lesions are frequently undetectable on CT; therefore, MRI is the imaging of choice and imperative for correct diagnosis. Early identification and treatment can reduce the risk of worsening ischemia and increase the probability of complete recovery. Medical education programs and emergency departments should include teaching and raising awareness of uncommon cerebrovascular accident presentations to improve patient outcomes.
